# Swine acute diarrhea syndrome coronavirus Nsp1 suppresses IFN-λ1 production by degrading IRF1 via ubiquitin–proteasome pathway

**DOI:** 10.1186/s13567-024-01299-6

**Published:** 2024-04-08

**Authors:** Chunhui Zhong, Gaoli She, Yukun Zhao, Yufang Liu, Jingmin Li, Xiaona Wei, Zexin Chen, Keyu Zhao, Zhiqing Zhao, Zhichao Xu, Hao Zhang, Yongchang Cao, Chunyi Xue

**Affiliations:** 1 State Key Laboratory of Biocontrol, School of Life Sciences, Sun Yat-Sen University, Guangzhou, China; 2 School of Life Sciences, Zhengzhou University, Zhengzhou, Henan China

**Keywords:** Swine acute diarrhea syndrome coronavirus, Nsp1, IFN-λ, IRF1, innate immune evasion

## Abstract

Swine acute diarrhea syndrome coronavirus (SADS-CoV) is a novel porcine enteric coronavirus that causes acute watery diarrhea, vomiting, and dehydration in newborn piglets. The type III interferon (IFN-λ) response serves as the primary defense against viruses that replicate in intestinal epithelial cells. However, there is currently no information available on how SADS-CoV modulates the production of IFN-λ. In this study, we utilized IPI-FX cells (a cell line of porcine ileum epithelium) as an in vitro model to investigate the potential immune evasion strategies employed by SADS-CoV against the IFN-λ response. Our results showed that SADS-CoV infection suppressed the production of IFN-λ1 induced by poly(I:C). Through screening SADS-CoV-encoded proteins, nsp1, nsp5, nsp10, nsp12, nsp16, E, S1, and S2 were identified as antagonists of IFN-λ1 production. Specifically, SADS-CoV nsp1 impeded the activation of the IFN-λ1 promoter mediated by MAVS, TBK1, IKKε, and IRF1. Both SADS-CoV and nsp1 obstructed poly(I:C)-induced nuclear translocation of IRF1. Moreover, SADS-CoV nsp1 degraded IRF1 via the ubiquitin-mediated proteasome pathway without interacting with it. Overall, our study provides the first evidence that SADS-CoV inhibits the type III IFN response, shedding light on the molecular mechanisms employed by SADS-CoV to evade the host immune response.

**Supplementary Information:**

The online version contains supplementary material available at 10.1186/s13567-024-01299-6.

## Introduction

Coronaviruses (CoVs) are classified into four genera: α-, β-, γ-, and *δ*-CoVs, in which the first two predominantly originate in bats and infect other mammals [1]. In 2017, a novel bat-HKU2-like porcine α-CoV, known as swine acute diarrhea syndrome coronavirus (SADS-CoV), was first identified in Guangdong province, China. It mainly affects the intestine and causes severe watery diarrhea in newborn piglets, resulting in a fatal mortality rate of nearly 90% [2–6].

SADS-CoV possesses a large single-stranded positive-sense RNA genome, approximately 27 000 nucleotides in length, with a 5′ cap and a 3′ polyadenylated tail [3, 5, 7]. The 5′ two-thirds of the genome, containing ORF1a and ORF1b, encodes the functionally conserved replicase-transcriptase, which is composed of 16 nonstructural proteins (nsp1-16). The remaining 3′ one-third of the genome encodes three accessory proteins (NS3a, NS7a, and NS7b), as well as four structural proteins: spike (S), envelope (E), membrane (M), and nucleocapsid (N) proteins [3, 5, 7]. Among these proteins, nsp1 is encoded only in α- and β-CoVs and represents the first mature viral protein expressed in the host cell cytoplasm [8]. Structural analyses have revealed a high degree of similarity in the core structure of nsp1 among CoVs [9, 10]. It is considered as an essential virulence determinant, employing various strategies to inhibit host gene expression and facilitate the evasion of host immunity by suppressing IFN induction, signaling, and production [11–13].

Mammalian innate immunity serves as a crucial defense against viral infection, and the IFN system plays a pivotal role in the host innate immune response [14]. During viral replication, double-stranded RNAs, known as pathogen-associated molecular patterns (PAMPs), can be recognized by host pattern-recognition receptors (PRRs), such as retinoic acid-induced gene I (RIG-I) or melanoma differentiation gene 5 (MDA5). Upon binding viral ligands that act as PAMPs, RIG-I/MDA5 recruit the mitochondrial antiviral-signaling (MAVS) protein, which activates transcriptional factors including interferon regulatory factors (IRFs) and NF-κB. These factors subsequently translocate to the nucleus and induce the production of type I and III IFNs [15]. While the induction processes and mechanisms of type I and III IFNs are similar, type I IFN receptors are expressed ubiquitously, while type III IFN (IFN-λ) receptors are primarily expressed on epithelial cells. This suggests that IFN-λ plays a critical role in protecting epithelial surfaces against viral infections [16–21]. In the case of intestinal viruses, the IFN-λ response acts as the first line of defense [22, 23]. Several CoVs have been reported to interfere with innate immunity and delay IFN responses during infection [24–28].

IRF1, a member of the transcriptional regulator family, was the first recognized member of the IRF family [29]. It is expressed at low basal levels in cells and exhibits a high responsiveness to viral stimuli. Upon viral infection, IRF1 translocates to the nucleus, leading to the rapid activation of IFN response [30, 31]. Recent researches have highlighted the unique role of IRF1 in IFN-λ production [32–34]. As an IFN-stimulated gene (ISG), both the mRNA transcript and protein of IRF1 are short-lived, allowing the host to exert rapid and dynamic regulation in response to viral infection [35]. It has been observed that IRF1 undergoes rapid degradation through the ubiquitin–proteasome pathway and the rate of degradation can be modulated in response to cellular conditions and specific stresses [36, 37].

Previous studies have indicated that SADS-CoV infection can inhibit the type I IFN response as a strategy to evade the host innate immunity [10, 24, 38, 39]. However, it remains unclear whether SADS-CoV also suppresses the type III IFN response, which is the primary defense mechanism against intestinal viruses. In this study, we conducted experiments to demonstrate that SADS-CoV infection significantly suppressed the production of IFN-λ1 induced by poly(I:C), and nsp1 was identified as a potent antagonist of IFN-λ1. Moreover, SADS-CoV nsp1 obstructed the nuclear translocation of IRF1 and targeted IRF1 for degradation through the ubiquitin-mediated proteasome pathway. These findings offer new insights into the mechanisms employed by SADS-CoV to evade the host innate immune response.

## Materials and methods

### Virus and cells

SADS-CoV strain GDS04 (GenBank accession number: MF167434.1) was isolated and propagated by our laboratory [2, 6, 40]. The IPI-FX cell line was derived from porcine ileum epithelium. HEK-293T cells were preserved in our laboratory. Both cell lines were cultured in Dulbecco’s modified Eagle’s medium (DMEM) supplemented with 10% heat-inactivated fetal bovine serum (Thermo Fisher), 100 U/mL penicillin, and 10 μg/mL streptomycin sulfate at 37 °C with 5% CO_2_ in a humidified incubator.

### Reagents and antibodies

Polyinosinic-polycytidylic acid [poly(I:C)] used as the positive control was purchased from InvivoGen (San Diego, CA). The Dual-Luciferase® Reporter Assay System was purchased from Promega (Madison, WI). Mouse anti-SADS-CoV N polyclonal antibody (pAb) [1:1000 for indirect-immunofluorescence assay (IFA), 1:1000 for Western blot (WB), and 1:200 for Confocal] was prepared by our laboratory. Mouse anti-GFP monoclonal antibody (mAb) (1:1000 for WB, and 1:200 for confocal), and rabbit anti-IRF1 pAb (1:1000 for WB, and 1:500 for confocal) were purchased from Santa Cruz Biotechnology (Dallas, TX). Mouse anti-HA mAb (1:3000 for WB, and 1:50 for Co-IP) was purchased from Abmart (Berkeley Heights, NJ). Mouse anti-GAPDH mAb (1:1000 for WB), rabbit anti-HA pAb (4 μg for IP), fluorescein (FITC)–conjugated goat anti-mouse IgG, and horseradish peroxidase (HRP)-conjugated goat anti-mouse/rabbit IgG were purchased from Proteintech (Rosemont, IL, USA). Mouse anti-FLAG mAb, Alexa Fluor 594-conjugated (goat anti-rabbit), Alexa Fluor 488/647-conjugated (goat anti-mouse) secondary antibodies, and MG132 were purchased from Sigma (St. Louis, MO, USA). Chloroquine (CQ) was purchased from MedChemExpress (MCE, Monmouth Junction, NJ, USA). Cycloheximide (CHX) was purchased from Cayman Chemical (Ann Arbor, MI, USA).

### Plasmids

The individual genes of SADS-CoV were cloned into pEGFP-N1 with an EGFP-tag at the C-terminus. SADS-CoV nsp1 was cloned into pcDNA3.1 vector with a FLAG-tag at the C-terminus. The porcine reporter plasmid IFN-λ1-luc was constructed using pGL3-basic vector. The porcine *MAVS* and *IRF1* genes were cloned into pcDNA3.1 vector with a HA-tag at the N-terminus. The internal control plasmid pRL-TK was preserved by our laboratory.

### Indirect-immunofluorescence assay and confocal microscopy

Cells were fixed with 4% paraformaldehyde for 15 min at room temperature (RT) and permeabilized with 0.1% Triton X-100 for 15 min at RT. After three washes with phosphate-buffered saline (PBS), the cells were incubated with 1% bovine serum albumin (BSA) for 1 h at 37 °C, followed by incubation with the primary antibody for 1 h at 37 °C, and then a fluorochrome-conjugated secondary antibody in the dark for 1 h at 37 °C. The cell nuclei were stained with 4′, 6-diamidino-2-phenylindole (DAPI) for 8 min. After three washes, the coverslips containing the stained cells were mounted onto the microscope slides using Fluoromount-G mounting medium (Beyotime, Shanghai, China). The fluorescence was visualized using a Nikon Ti microscope and a Leica TCS-SP5 confocal fluorescence microscope.

### Western blot analysis

Cells were washed twice with precooled PBS and lysed with a cell lysis buffer for Western and IP (Beyotime) with a protease inhibitor cocktail (MCE). The cell lysates were separated by 12.5% SDS-PAGE and electro-transferred to a PVDF membrane (Millipore). The membranes were blocked with 5% nonfat dry milk in TBST for 1 h and incubated with the primary antibody at 4 °C overnight. After three washes, the membranes were incubated with HRP-conjugated secondary antibody for 1 h at RT. After three washes, the protein blots were visualized using an enhanced chemiluminescence (ECL) detection system (NMC Biotech) and the chemiluminescent signals emitted from the protein blots were captured using the GelView 6000 Pro imaging system according to the manufacturer’s instructions (BLT, Guangdong, China).

### RNA isolation and RT-qPCR

Cells were washed once with PBS and lysed with lysis buffer. Total cellular RNA was extracted using a RNA isolation kit according to the manufacturer’s instructions (EZBioscience, Roseville, MN) and reverse transcribed to cDNA using ReverTra Ace® qPCR RT master mix with gDNA remover (Toyobo, Osaka, Japan). The synthesized cDNA was subjected to real-time quantitative PCR using PerfectStart™ Green qPCR SuperMix (TransGen, Beijing, China) at least triplicate with a Light Cycler 480 real-time PCR system (Roche, Mannheim, Germany). The RT-qPCR primers are listed in the Additional file 1. The GAPDH gene was used as an internal control for each experiment. The relative transcription levels of the target genes are presented as fold changes relative to the respective controls using the 2^−ΔΔCT^ method.

### Dual-reporter assay

To investigate the effect of SADS-CoV infection on poly(I:C)-induced IFN-λ1 promoter activity, IPI-FX cells were cultured in 24-well plates until they reached 80% confluency. Cells were then transfected with the indicated luciferase reporter plasmid and pRL-TK at a ratio of 1:0.01 using the jetPRIME® transfection reagent, following the manufacturer’s instructions (Polyplus-transfection, Illkirch, France). 12 h post-transfection, the cells were infected with SADS-CoV at a MOI of 1 for 12 h, followed by stimulation with poly(I:C) for an additional 12 h.

To identify the viral antagonists of IFN-λ1 production, cells were transfected with a plasmid expressing individual viral proteins, along with the luciferase reporter plasmid and pRL-TK at a ratio of 1:1:0.01 for 24 h, followed by stimulation with poly(I:C) for 12 h.

To determine the stage at which nsp1 exerts its inhibitory activity in the RLR pathway, cells were transfected with the expression plasmids of nsp1 and MAVS/TBK1/IKKε/IRF1, along with the luciferase reporter plasmid and pRL-TK at a ratio of 1:1:0.1:0.01 for 24 h.

After the respective treatments, the cells were lysed using 1 × passive lysis buffer for 15 min at RT, and the firefly and renilla luciferase activities were measured using a GloMax-20/2 luminometer with a Dual-Luciferase® reporter assay system (Promega). Data are presented as the relative firefly luciferase activity normalized to the renilla luciferase activity from three independent experiments.

### Co-immunoprecipitation analysis

Cells were washed twice with precooled PBS and lysed using cell lysis buffer for WB and IP (Beyotime) with a protease inhibitor cocktail. The cell lysates were subjected to immunoprecipitation using protein A/G magnetic beads (Beyotime) overnight at 4 °C. The beads were then incubated with the indicated antibody for 4 h at 4 °C. After five washes with lysis buffer, the protein A/G magnetic beads were mixed with 40 μL of 1 $\times$ SADS-PAGE sample loading buffer (FUDE Science and Technology, Shandong, China) and boiled for 10 min at 100 °C. The immunoprecipitates were then analyzed by WB.

### Statistical analysis

All data are shown as the mean ± standard deviation (SD) of three independent experiments. Statistical analyses were conducted by using GraphPad Prism 8 for the T-test. Asterisks in figures indicate statistical significance as follows: *, *p* < 0.05, **, *p* < 0.01, ***, *p* < 0.001.

## Results

### SADS-CoV efficiently infects IPI-FX cells

**Figure 1:**
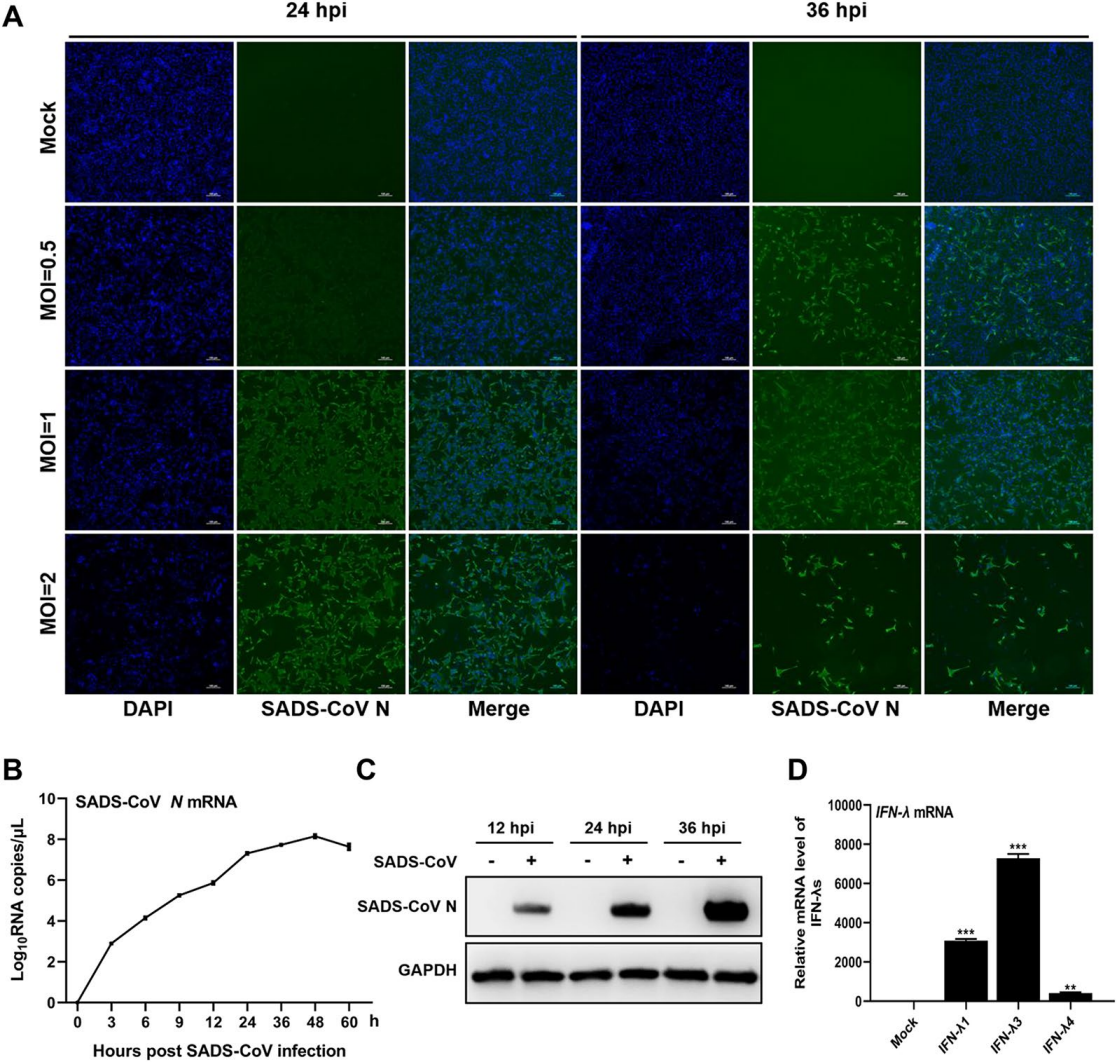
**SADS-CoV efficiently infects IPI-FX cells**. **A**\ Expression of SADS-CoV N protein in infected-cells detected by immunofluorescence assay. IPI-FX cells were either mock-infected or infected with SADS-CoV at different multiplicities of infection (MOI = 0.5, 1, 2). At 24 and 36 hpi, cells were fixed and incubated with a polyclonal antibody against SADS-CoV N protein (green). Scale bar = 100 μm. **B** Expression of SADS-CoV *N* mRNA in infected-cells detected by RT-qPCR. IPI-FX cells were either mock-infected or infected with SADS-CoV at a MOI of 1. The mRNA level of the SADS-CoV *N* gene was measured at the indicated hours post-infection using RT-qPCR. **C** Expression of SADS-CoV N protein in infected cells detected by Western blot. IPI-FX cells were either mock-infected or infected with SADS-CoV at a MOI of 1. Cell extracts were prepared at 12, 24, and 36 hpi and subjected to Western blot analysis. **D** Induction of IFN-λ in IPI-FX cells after poly(I:C) stimulation. Cells were transfected with or without 1 μg/mL poly(I:C). At 12 hpi, the mRNA levels of the *IFN-λ1*, *IFN-λ3*, and *IFN-λ4* genes were measured using RT-qPCR. The mRNA levels of IFN-λ after poly(I:C) stimulation were normalized to those of individual IFN-λ without treatment. Data are represented as the mean ± SD of three replicates. **P < 0.01; ***P < 0.001.

SADS-CoV isolated from clinical samples has poor adaptability to cells in vitro. In this study, the SADS-CoV GDS04 P15 strain, which has been shown to cause cytopathic effects in Vero cells and has a high mortality rate of 87.5% in newborn piglets [40], was used for in vitro research. The primary target cells of SADS-CoV in vivo are porcine intestinal epithelial cells [2, 3, 5, 6], so we investigated the replication and proliferation of SADS-CoV in IPI-FX cells, which are derived from porcine ileum epithelial cells. To assess the efficiency of SADS-CoV infection in IPI-FX cells, cells were mock-infected or infected with SADS-CoV at a MOI of 0.5, 1, and 2. At 24 and 36 h post-infection (hpi), the expression of SADS-CoV N protein was detected using IFA. As shown in Figure 1A, specific fluorescence was observed in SADS-CoV-infected IPI-FX cells at 24 hpi. As the infection titer or time increased, the cytopathic effect became more pronounced, with more detached cells, indicating efficient replication of SADS-CoV in IPI-FX cells. To examine the expression of SADS-CoV *N* mRNA, IPI-FX cells were infected with SADS-CoV at a MOI of 1, and the mRNA level of the *N* gene at different time points was measured by RT-qPCR. The replication curve of the *N* gene demonstrated productive replication of SADS-CoV (Figure 1B). WB analysis further confirmed the efficient infection of IPI-FX cells by SADS-CoV (Figure 1C).

To investigate whether IPI-FX cells are capable of producing IFN-λ, the transcription levels of different types of IFN-λ were measured using RT-qPCR. As shown in Figure 1D, after stimulation with poly(I:C), the transcription levels of IFN-λ1, IFN-λ3, and IFN-λ4 in IPI-FX cells increased significantly by approximately 3000-fold, 7000-fold, and 500-fold, respectively. This indicates that IPI-FX cells are capable of efficiently producing IFN-λ. Based on these findings, we can conclude that IPI-FX cells are susceptible to SADS-CoV infection and can express IFN-λ. This makes IPI-FX cells a suitable model for studying the type III IFN response, which may be regulated by SADS-CoV.

### SADS-CoV suppresses poly(I:C)-induced IFN-λ1 production

**Figure 2:**
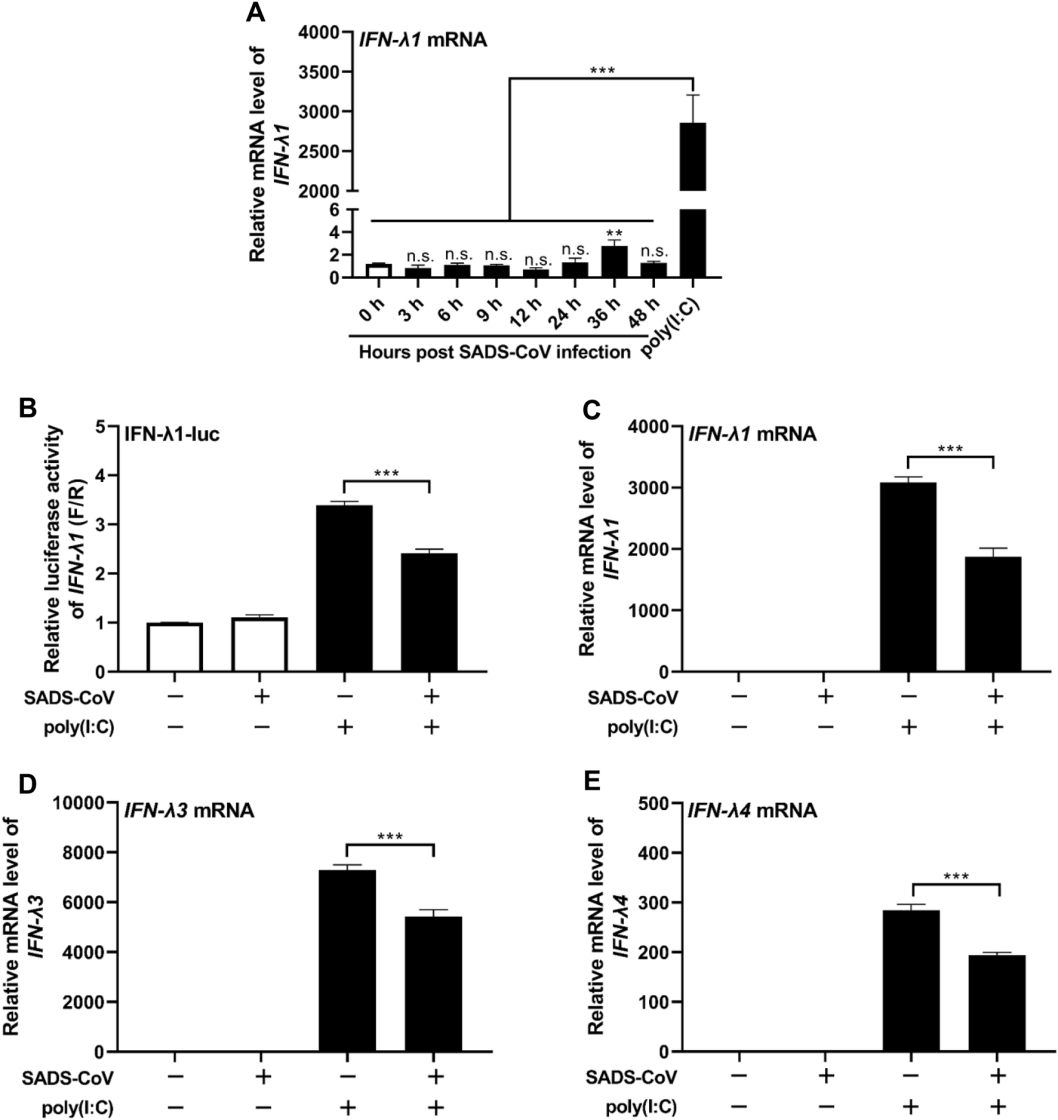
**SADS-CoV suppresses poly(I:C)-induced IFN-λ1 production.** **A** IFN-λ1 mRNA expression in SADS-CoV-infected cells. IPI-FX cells were either mock-infected or infected with SADS-CoV at a MOI of 1. The mRNA level of the *IFN-λ1* gene was measured at the indicated hours post-infection using RT-qPCR. Mock-infected cells stimulated with 1 μg/mL poly(I:C) for 12 h were used as the positive control. **B** SADS-CoV suppresses poly(I:C)-induced IFN-λ1 promoter activity. IPI-FX cells were co-transfected with IFN-λ1-luc and pRL-TK at a ratio of 1:0.1 for 12 h. Then, cells were either mock-infected or infected with SADS-CoV at a MOI of 1 for 12 h. Subsequently, cells were transfected with or without poly(I:C) for an additional 12 h, and the relative activity of the IFN-λ1 promoter was determined using a luciferase reporter assay. **C**–**E** SADS-CoV suppresses poly(I:C)-induced IFN-λ1 (**C**), IFN-λ3 (**D**), and IFN-λ4 (**E**) mRNA levels. IPI-FX cells were infected or mock-infected with SADS-CoV at a MOI of 1 for 12 h and then transfected with or without 1 μg/mL poly(I:C) for an additional 12 h. Total RNA was extracted, and the relative mRNA expression of IFN-λ1, IFN-λ3, and IFN-λ4 was determined using RT-qPCR. Data are represented as the mean ± SD of three replicates. ***P < 0.001.

To investigate whether SADS-CoV infection can induce IFN-λ production in IPI-FX cells, cells were infected with SADS-CoV at a MOI of 1, and cell lysates were collected at different time points post-infection for RT-qPCR analysis. As shown in Figure 2A, SADS-CoV infection only slightly induced the expression of IFN-λ1 mRNA at 36 hpi, which was then downregulated. In contrast, transfection with poly(I:C) as a positive control resulted in a remarkable induction of IFN-λ1 mRNA expression. However, the mRNA expression of IFN-λ3 and IFN-λ4 was not detected at any of the examined time points in SADS-CoV-infected cells (data not shown). To determine whether SADS-CoV infection suppressed poly(I:C)-induced IFN-λ expression, cells were transfected with IFN-λ1-luc/pRL-TK, infected with SADS-CoV, and then stimulated with poly(I:C). The relative activity of the IFN-λ1 promoter was assessed using a luciferase reporter assay. As shown in Figure 2B, the luciferase activity was barely detectable in SADS-CoV-infected cells, and the activation of the IFN-λ1 promoter induced by poly(I:C) was significantly inhibited compared to mock-infected cells.

Furthermore, RT-qPCR was conducted to detect IFN-λ1 transcription in SADS-CoV-infected, poly(I:C)-stimulated cells. As shown in Figure 2C, the expression of IFN-λ1 mRNA induced by poly(I:C) was significantly inhibited in SADS-CoV-infected cells compared to mock-infected cells. Similarly, the mRNA expression of IFN-λ3 and IFN-λ4 induced by poly(I:C) were also noticeably blocked in SADS-CoV-infected cells compared to mock-infected cells (Figures 2D and E). These results suggest that SADS-CoV inhibits poly(I:C)-induced IFN-λ production in IPI-FX cells.

### Identification of viral antagonists of IFN-λ1 production

**Figure 3:**
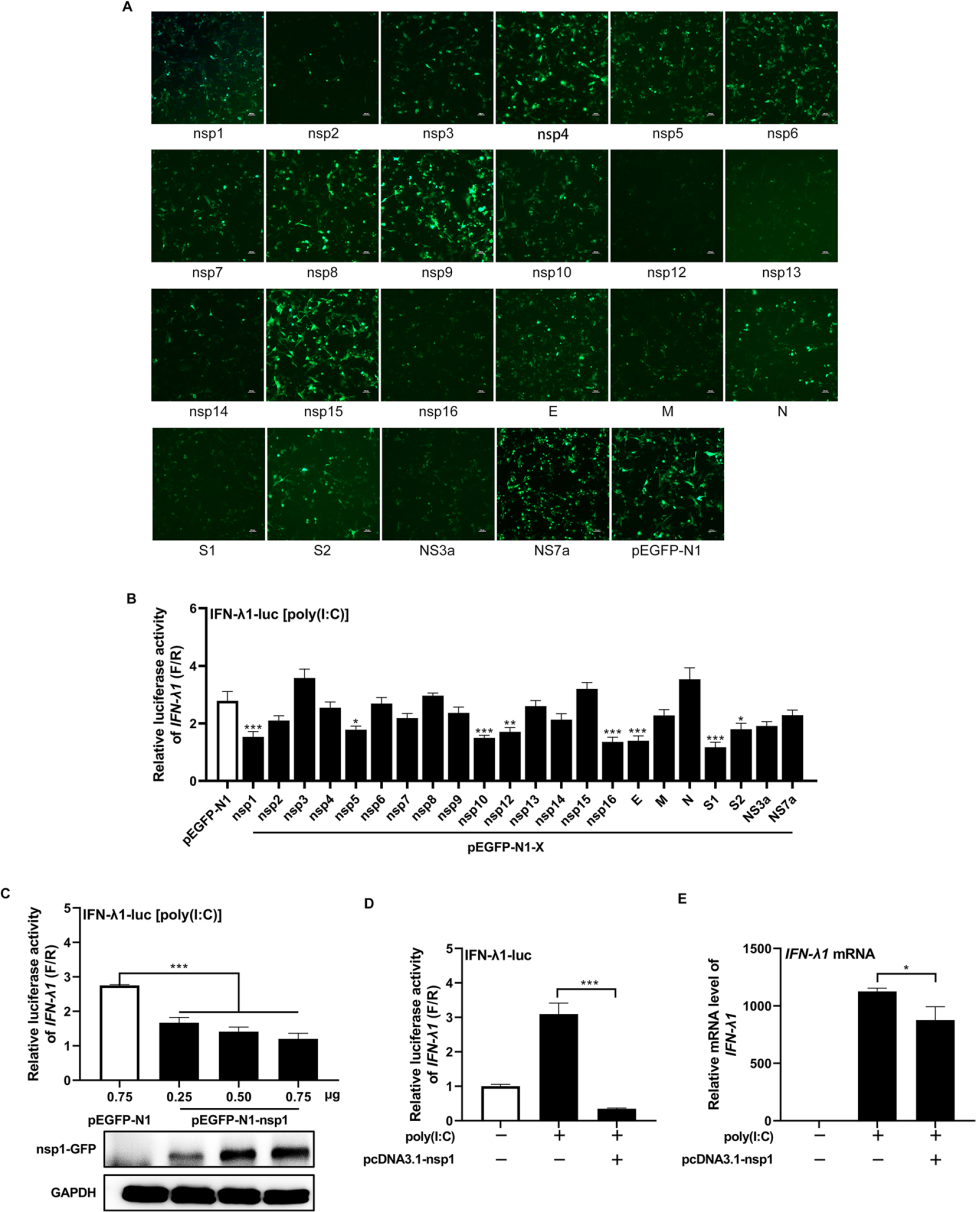
**Identification of SADS-CoV proteins antagonizing IFN-λ1.** **A** Expression of individual SADS-CoV proteins in IPI-FX cells detected by IFA. **B** Suppression of IFN-λ1 promoter activity by individual SADS-CoV proteins. IPI-FX cells were co-transfected with plasmids expressing individual SADS-CoV proteins, IFN-λ1-luc and pRL-TK at a ratio of 1:0.1:0.01. At 24 h post-transfection, cells were transfected with or without 1 μg/mL poly(I:C) for 12 h. Cell lysates were prepared, and luciferase activity was measured. The IFN-λ1 promoter activity induced by poly(I:C) stimulation was normalized to that without stimulation. **C** Dose-dependent inhibition of poly(I:C)-induced IFN-λ1 promoter activity by SADS-CoV nsp1. IPI-FX cells were transfected with different amounts (0.25/0.5/0.75 μg) of pEGFP-nsp1 or control plasmid pEGFP-N1, along with IFN-λ1-luc and pRL-TK at a ratio of 1:0.1:0.01 for 24 h. Subsequently, cells were transfected with or without 1 μg/mL poly(I:C) for 12 h. Cell lysates were prepared, and luciferase activity was measured. The IFN-λ1 promoter activity induced by poly(I:C) stimulation was normalized to that without stimulation. **D** Suppression of poly(I:C)-induced IFN-λ1 promoter activity by SADS-CoV nsp1. IPI-FX cells were transfected with pcDNA3.1-nsp1 or control plasmid pcDNA3.1, along with IFN-λ1-luc and pRL-TK at a ratio of 1:0.1:0.01 for 24 h. Subsequently, cells were transfected with or without 1 μg/mL poly(I:C) for 12 h. Cell lysates were prepared, and luciferase activity was measured. **E** Suppression of poly(I:C)-induced IFN-λ1 mRNA expression by SADS-CoV nsp1. IPI-FX cells were transfected with pcDNA3.1-nsp1 or the control plasmid pcDNA3.1 for 24 h, followed by transfection with or without 1 μg/mL poly(I:C) for 12 h. Total cellular RNA was extracted, and the expression of IFN-λ1 mRNA was determined using RT-qPCR. *P < 0.05; **P < 0.01; ***P < 0.001.

The infection study demonstrated that IFN-λ1 inhibited the replication of SADS-CoV in IPI-FX cells (Additional file 2), highlighting the importance of IFN-λ1 in SADS-CoV infection. To identify which viral proteins are responsible for antagonizing IFN-λ1 production, the expression of 22 individual SADS-CoV proteins were confirmed using IFA (Figure 3A), and SADS-CoV proteins were screened for their ability to suppress the activity of the IFN-λ1 promoter using a reporter assay. IPI-FX cells were co-transfected with plasmids expressing individual viral proteins along with luciferase reporter plasmids. Cells were then stimulated with poly(I:C), and a luciferase reporter assay was performed. As shown in Figure 3B, several viral nonstructural proteins (nsp1, nsp5, nsp10, nsp12, and nsp16) were found to downregulate IFN-λ1 promoter activity. Among the structural proteins, E, S1, and S2 were identified as suppressors of IFN-λ1 induction.

Of these viral antagonists of IFN-λ1 production, nsp1 exhibited a significant inhibitory effect (*P* < 0.001, Figure 3B). Nsp1 is known as a major virulence factor and a multifunctional viral antagonist for the innate immune response in coronavirus [11–13, 41]. Therefore, nsp1 was selected for further investigation. To confirm the screening results, increasing doses (0.25/0.5/0.75 μg) of a plasmid encoding nsp1, along with IFN-λ1-luc and pRL-TK plasmids, were co-transfected into IPI-FX cells. Cells were then stimulated with poly(I:C), and a luciferase reporter assay was conducted. As shown in Figure 3C, the luciferase activity decreased as the amount of nsp1 increased, indicating a dose-dependent inhibitory effect of nsp1 on IFN-λ1 promoter activation induced by poly(I:C). To further confirm the inhibition of IFN-λ1 by nsp1, the *nsp1* gene was inserted into the pcDNA3.1 vector, and a luciferase reporter assay and RT-qPCR were performed. As shown in Figures 3D and E, nsp1 inhibited poly(I:C)-induced IFN-λ1 promotor activity and mRNA expression. Taken together, these findings suggest that SADS-CoV encodes multiple antagonists to suppress IFN-λ1 induction, and nsp1 is identified as a potent viral antagonist that inhibits IFN-λ1 production in a dose-dependent manner.

### SADS-CoV nsp1 inhibits the RLR signaling pathway

**Figure 4:**
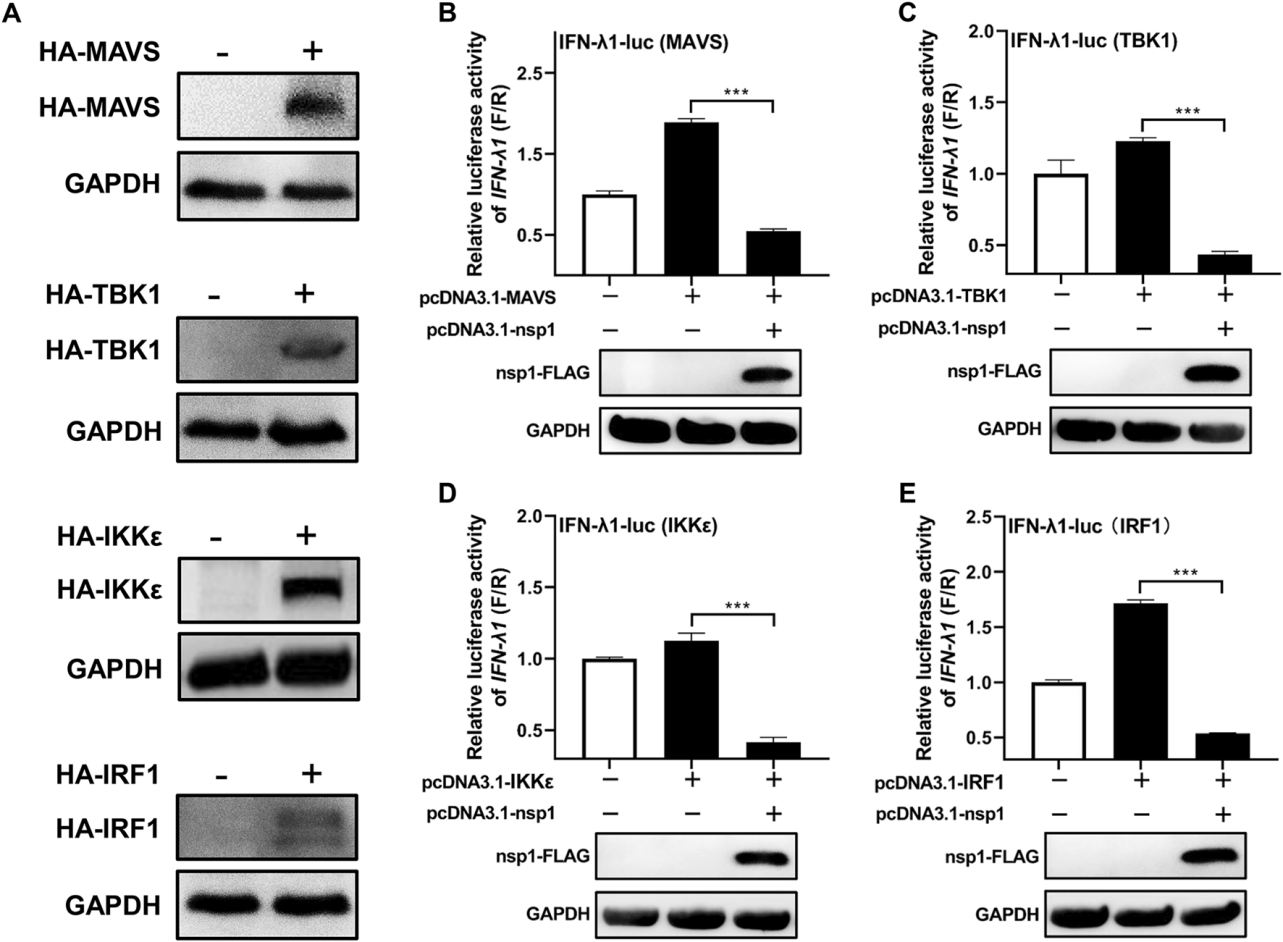
**Inhibition of the RLR signaling pathway by SADS-CoV nsp1**. **A** Expression of MAVS, TBK1, IKKε, and IRF1 in IPI-FX cells detected by Western blot. **B**–**E** SADS-CoV nsp1 inhibits the RLR signaling pathway. IPI-FX cells were co-transfected with nsp1, along with expression plasmids for MAVS (**B**), TBK1 (**C**), IKKε (**D**), or IRF1 (**E**), as well as IFN-λ1-luc and pRL-TK at a ratio of 1:1:0.1:0.01 for 24 h. Cell lysates were prepared, and luciferase activity was measured. Data are represented as the mean ± SD of three replicates. ***P < 0.001.

To further investigate the specific target of SADS-CoV nsp1 in antagonizing IFN-λ1 production, several key molecules in the RLR signaling pathway, including MAVS, TBK1, IKKε, and IRF1, were assessed using a reporter assay. Cells were co-transfected with plasmids expressing these crucial molecules, the nsp1 expression plasmid, and the luciferase reporter plasmid. The reporter activity was then measured. As shown in Figure 4, nsp1 reduced the activation of IFN-λ1 promoter activity mediated by MAVS, TBK1, IKKε, and IRF1. This indicates that nsp1 inhibits the IFN-λ1 production pathway by targeting IRF1 or its associated molecules.

### SADS-CoV and nsp1 block IRF1 nuclear translocation stimulated by poly(I:C)

**Figure 5:**
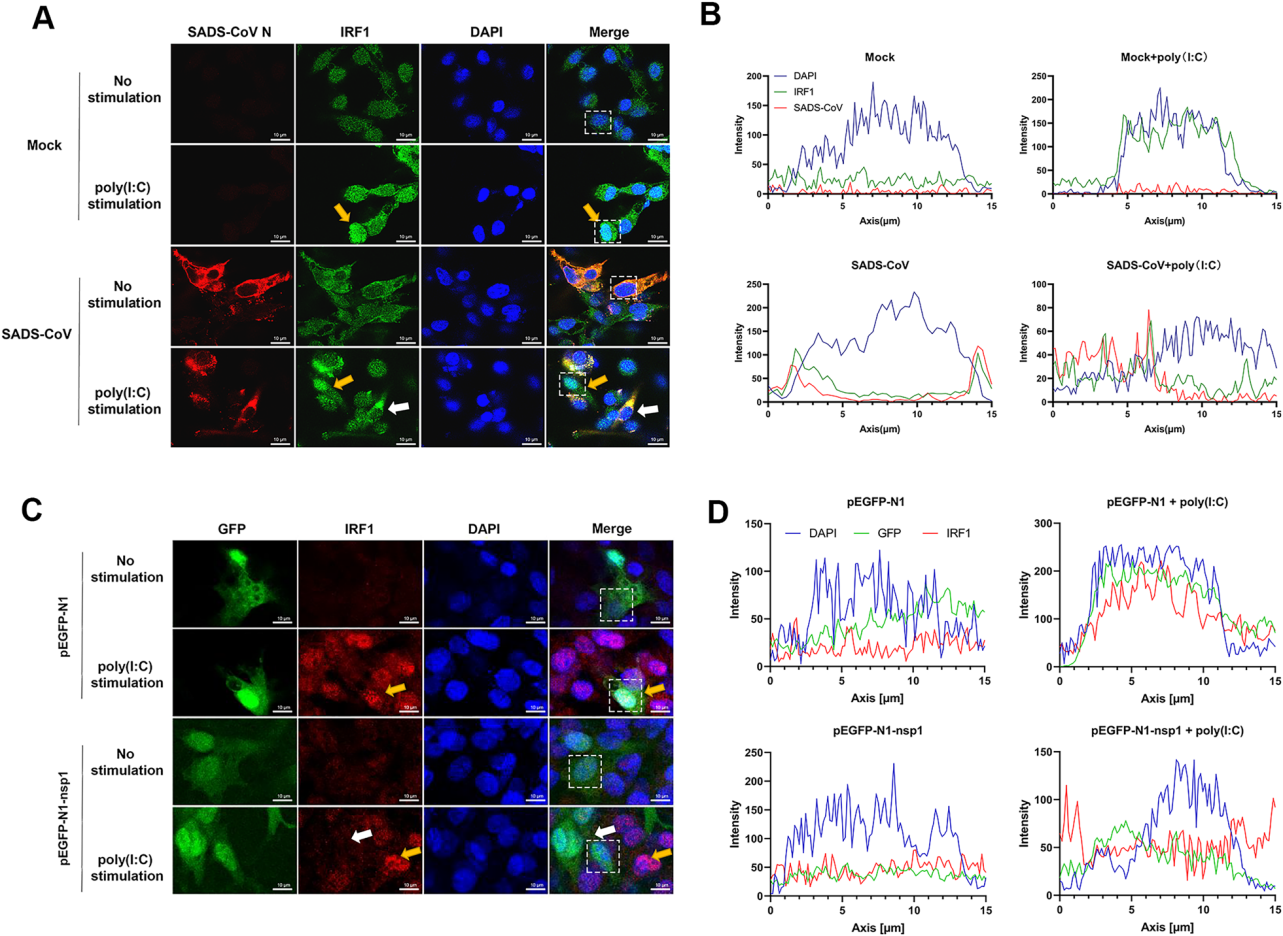
**SADS-CoV and nsp1 inhibit the nuclear translocation of IRF1 induced by poly(I:C) stimulation**. **A** SADS-CoV blocks IRF1 nuclear translocation stimulated by poly(I:C). IPI-FX cells were either infected with SADS-CoV (MOI = 1) or mock-infected for 12 h. Subsequently, cells were transfected with or without 1 μg/mL poly(I:C) for an additional 12 h. After fixation, cells were stained with anti-SADS-CoV N antibody and anti-IRF1 antibody. In the images, yellow arrows indicate IRF1 in the nucleus, while white arrows indicate IRF1 in the cytoplasm. Bar = 10 μm. **B** Intensity profiles of SADS-CoV (red), IRF1 (green), and DAPI (blue) calculated from a white box in the image. **C** SADS-CoV nsp1 blocks IRF1 nuclear translocation stimulated by poly(I:C). IPI-FX cells were transfected with either pEGFP-nsp1 or control plasmid pEGFP-N1 for 24 h. Then, cells were transfected with or without 1 μg/mL poly(I:C) for 12 h. After fixation, cells were stained with anti-IRF1 antibody. Bar = 5 μm. **D** Intensity profiles of nsp1 (green), IRF1 (red), and DAPI (blue) calculated from a white box in the image.

Based on previous studies, it is known that IRF1 is an interferon regulator that plays an important role in inducing IFN-λ production [23, 27, 32, 42, 43]. When activated, IRF1 translocates to the nucleus, leading to the expression of IFN-λ [44]. Based on these findings, we speculated that SADS-CoV nsp1 might target IRF1 to reduce IFN-λ production. To investigate whether SADS-CoV infection affects the nuclear translocation of IRF1, cells were infected with SADS-CoV and stimulated with poly(I:C), followed by antibody staining for IRF1. The confocal images of the different groups are shown in Figure 5A, and the fluorescence intensity is shown in Figure 5B. In the presence of poly(I:C) stimulation, IRF1 translocated to the nucleus (second panel, yellow arrow). However, in SADS-CoV-infected cells, IRF1 remained in the cytoplasm (bottom panel, white arrow) even after poly(I:C) stimulation. This indicates that SADS-CoV infection blocked the poly(I:C)-induced nuclear translocation of IRF1.

To further examine whether nsp1 is responsible for blocking IRF1 nuclear translocation, cells were transfected with either a vector or the nsp1 expression plasmid, followed by poly(I:C) stimulation. The confocal images of the different groups are shown in Figure 5C, and the fluorescence intensity is shown in Figure 5D. In cells expressing the vector, IRF1 was predominantly distributed in the cytoplasm, but poly(I:C) stimulation led to its translocation to the nucleus (second panel, yellow arrow). However, in cells expressing nsp1, IRF1 diffused throughout the cells (bottom panel, white arrow) even after poly(I:C) stimulation. This indicates that nsp1 blocked the poly(I:C)-induced nuclear translocation of IRF1.

### SADS-CoV nsp1 degrades IRF1 through the ubiquitin–proteasome pathway

**Figure 6:**
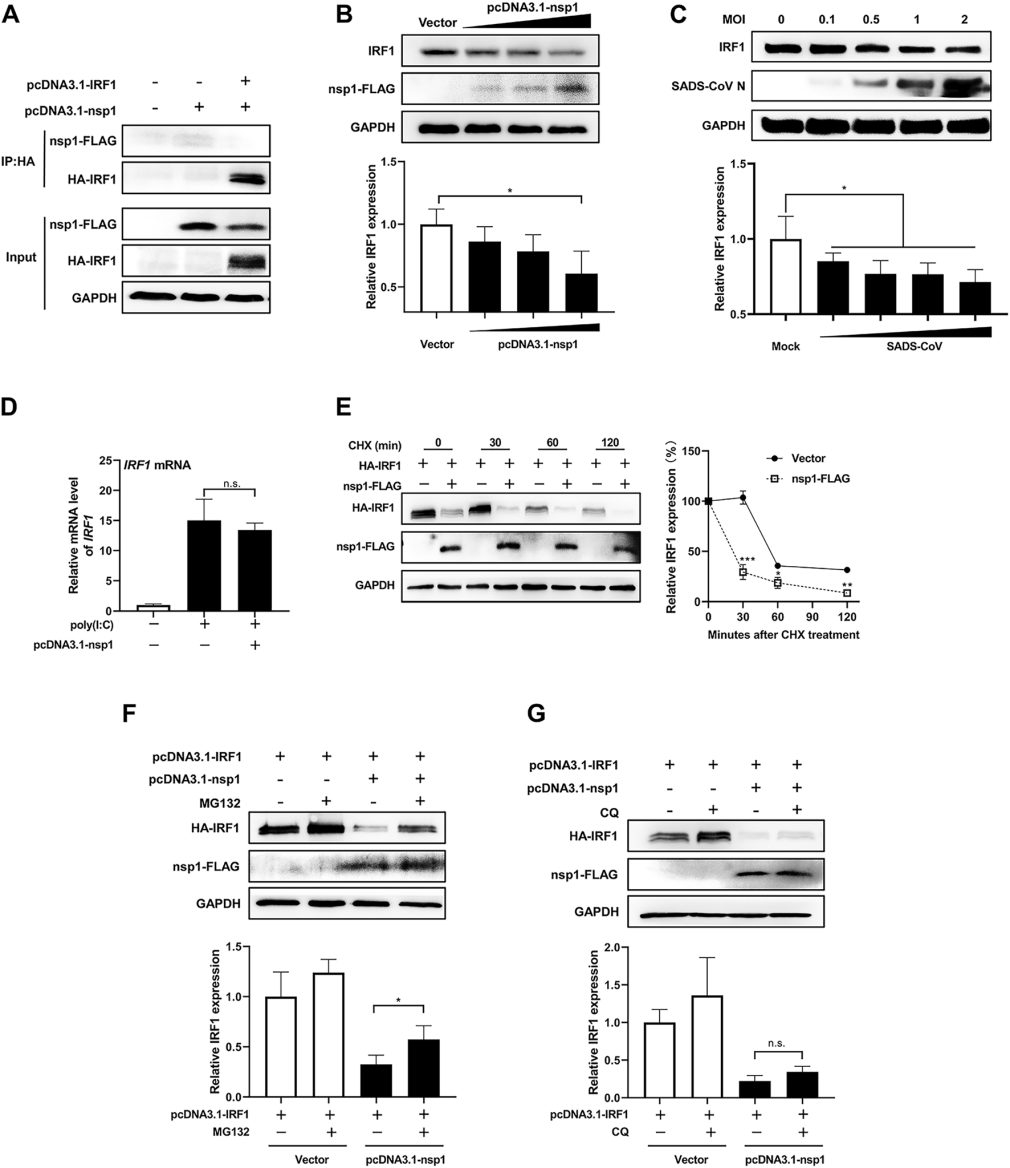
**SADS-CoV nsp1 degrades IRF1 through the ubiquitin–proteasome pathway.** **A** SADS-CoV nsp1 does not directly interact with IRF1. IPI-FX cells were co-transfected with pcDNA3.1-HA-IRF1 and pcDNA3.1-nsp1-FLAG for 24 h. Cell extracts were prepared and subjected to Co-IP analysis. **B** Endogenous IRF1 protein abundance is reduced by SADS-CoV nsp1. IPI-FX cells were transfected with pcDNA3.1 or pcDNA3.1-nsp1 for 24 h. Cell extracts were prepared and subjected to Western blot analysis. **C** Endogenous IRF1 protein abundance is reduced by SADS-CoV. IPI-FX cells were mock-infected or infected with SADS-CoV at a MOI of 1 for 24 h. Cell extracts were prepared and subjected to Western blot analysis. **D** SADS-CoV nsp1 does not regulate the expression of IRF1 at the transcriptional level. IPI-FX cells were transfected with pcDNA3.1 or pcDNA3.1-nsp1 for 24 h, and with or without 1 μg/mL poly(I:C) for 12 h. Total cellular RNA was prepared to detect IRF1 mRNA levels by RT-qPCR. **E** SADS-CoV nsp1 shortens the half-life of IRF1. 293-T cells were co-transfected with pcDNA3.1-HA-IRF1 and pcDNA3.1-nsp1-FLAG for 24 h, and treated with or without 25 μg/mL CHX for 30/60/120 min. Cell extracts were prepared and subjected to Western blot analysis. **F** MG132 treatment blocks IRF1 degradation caused by nsp1. 293-T cells were co-transfected with pcDNA3.1-HA-IRF1 and pcDNA3.1-nsp1-FLAG for 24 h, and treated with 50 μM MG132 for 3 h. The equal amount of DMSO was used as a control. Cell extracts were prepared and subjected to Western blot analysis. **G** CQ treatment does not block IRF1 degradation caused by nsp1. 293-T cells were co-transfected with pcDNA3.1-HA-IRF1 and pcDNA3.1-nsp1-FLAG for 24 h, and treated with 50 μM CQ for 10 h. The equal amount of DMSO was used as a control. Cell extracts were prepared and subjected to Western blot analysis. The average intensity of the band of endogenous or exogenous IRF1 are normalized to GAPDH by Image J software.

To investigate whether nsp1 interacts with IRF1 to inhibit IRF1 nuclear translocation, cells were co-transfected with nsp1-FLAG and HA-IRF1 expression plasmids, and a Co-IP assay was performed. As shown in Figure 6A, nsp1 and IRF1 bands were detected in the whole-cell lysates, but the nsp1 band could not be detected when IRF1 was used as the bait protein. Similarly, the IRF1 band could not be detected when nsp1 was used as the bait protein (data not shown). This suggests that there is no direct interaction between nsp1 and IRF1.

Previous studies have shown that the nsp1 protein of porcine epidemic diarrhea virus (PEDV) reduces the abundance of host proteins to evade the host IFN response [45, 46]. To investigate whether SADS-CoV nsp1 reduces IRF1 protein abundance, cells were transfected with increasing amounts of the nsp1 expression plasmid, and WB analysis was performed. As shown in Figure 6B, as the amount of nsp1 increased, the level of endogenous IRF1 protein gradually decreased. Similarly, as shown in Figure 6B, when cells were infected with increasing MOIs of SADS-CoV, the abundance of endogenous IRF1 protein decreased gradually. These findings suggest that nsp1 reduces the abundance of the IRF1 protein without directly interacting with it.

To determine how SADS-CoV nsp1 reduces the abundance of IRF1 protein, the effect of nsp1 on IRF1 transcription was examined. As shown in Figure 6D, co-transfection of nsp1-FLAG and HA-IRF1 expression plasmids followed by poly(I:C) stimulation did not show any variation in IRF1 mRNA levels, indicating that nsp1 may not regulate the expression of IRF1 at the transcriptional level. To investigate whether nsp1 affects IRF1 protein stability, cells were co-transfected with nsp1-FLAG and HA-IRF1 expression plasmids and treated with the protein synthesis inhibitor CHX. As shown in Figure 6E, the densitometric quantification of WB results showed that in the presence of nsp1 expression, the decay of IRF1 was significantly accelerated, and the half-life of IRF1 was reduced from approximately 50 min to approximately 20 min.

Protein degradation in eukaryotes is primarily mediated by the ubiquitin–proteasome system (UPS) and the autophagy-lysosome pathway (ALP). To determine the mechanism by which nsp1 degrades IRF1, cells were co-transfected with nsp1-FLAG and HA-IRF1 expression plasmids and treated with the proteasome inhibitor MG132 or the lysosome inhibitor CQ. As shown in Figures 6F and G, MG132 treatment blocked IRF1 degradation caused by nsp1, while CQ treatment did not influence IRF1 degradation. This suggests that SADS-CoV nsp1 degrades IRF1 through the ubiquitin–proteasome pathway.

## Discussion

As a newly emerging virus, SADS-CoV caused the death of approximately 25 000 piglets when it first broke out in Guangdong province, resulting in economic losses for the pig industry [5]. The main target of the virus is the intestinal mucosal immune system of pigs, causing diarrhea, dehydration, and death in newborn piglets [2–6]. Due to the specific expression of receptors, type III IFN plays a crucial role in innate mucosal immunity [47]. Previous studies on the innate immune interaction of SADS-CoV with hosts have shown that SADS-CoV employs strategies to evade the type I IFN response [10, 24, 39], but whether it can evade the type III IFN response remains unclear. Our report demonstrates that SADS-CoV antagonizes the production of IFN-λ1, and the nsp1 protein functions by suppressing IFN-λ1 production through the targeting of IRF1 via ubiquitin-mediated proteasome degradation.

To study the effect of SADS-CoV on IFN-λ production, it is necessary to obtain a cell-adapted SADS-CoV strain and a cell line that is both susceptible to the virus and capable of producing IFN-λ. The SADS-CoV strain used in this study was the GDS04 P15 strain, obtained from a wild-type SADS-CoV strain after 15 consecutive passages in Vero cells. It was adapted to cell growth while retaining high pathogenicity in newborn piglets [40]. The cells used in this study were IPI-FX cells, derived from subcloning porcine ileum epithelial cells (IPI-2I) through limited serial dilutions, and were highly susceptible to various porcine CoVs [48]. We first confirmed that IPI-FX cells were highly susceptible to SADS-CoV. Upon stimulation of IPI-FX cells with poly(I:C), the transcription of three different subtypes of IFN-λ was effectively induced, and the transcription levels ranking as IFN-λ3 > IFN-λ1 > IFN-λ4, consistent with the results from porcine intestinal epithelial cells (IPEC) by Zhang et al. [43]. This indicated that IPI-FX cells were suitable for studying the interaction between SADS-CoV and the type III IFN response. Subsequently, we explored the changes of IFN-λ in SADS-CoV-infected IPI-FX cells. The results showed that, similar to PEDV, classical swine fever virus (CSFV) and porcine deltacoronavirus (PDCoV) [27, 42, 43], SADS-CoV failed to effectively induce IFN-λ1 production in IPI-FX cells and could inhibit poly(I:C)-induced IFN-λ1 production, suggesting a critical role of IFN-λ1 in the pathogenesis of these viruses.

To evade the host immune response, CoVs have been reported to primarily counteract the IFN response through viral proteins. In PEDV, Zhang et al. identified nsp1, nsp3, nsp7, nsp14, nsp15, nsp16, E, M, N, and ORF3 as IFN-β antagonists, and nsp1, nsp3, nsp5, nsp8, nsp14, nsp15, nsp16, E, M, N, and ORF3 as IFN-λ1 antagonists [43, 46]. In this study, we found that nsp1, nsp5, nsp10, nsp12, nsp16, E, S1, and S2 could antagonize the production of IFN-λ1. Among them, nsp1, nsp16, and E overlapped with the interferon antagonist proteins of PEDV, indicating that these proteins may play similar and conserved roles in evading host innate immunity across different CoVs. As the largest known RNA viruses, CoVs including SARS-CoV, SARS-CoV-2, MERS-CoV, and others employ multiple viral proteins to antagonize various steps in the production and signaling pathways of IFNs [49]. Further studies are needed to elucidate the sophisticated immune evasion strategies of CoVs compared to other viruses.

The nsp1 protein of CoVs employs various strategies to inhibit IFN induction, signaling, and production, thereby evading host immunity [11–13]. For example, MERS-CoV nsp1 suppresses IFN-β expression through mRNA degradation and translation inhibition [50]. SARS-CoV-2 nsp1 inhibits IFN-β production by targeting multiple components upstream and downstream of IRF3 [49]. PEDV nsp1 utilizes multiple mechanisms to suppress IFN production, including interrupting the enhanceosome assembly of IRF3 and CREB-binding protein [46], interfering with IκBα phosphorylation and degradation [45], reducing the number of peroxisomes and blocking IRF1 nuclear translocation [43]. In this study, we aimed to investigate the mechanism by which SADS-CoV nsp1 antagonizes IFN-λ1 production. We co-transfected nsp1 and signaling molecules in the RLR pathway into cells to identify the possible target of nsp1. Most studies have reported that viruses target MAVS or its downstream molecules to antagonize IFN-λ production. For instance, rotavirus VP3 colocalizes with MAVS, leading to its degradation [51]. Zika virus NS5 targets IKK to inhibit IFN-λ1 promoter activation [52]. SARS-CoV-2 M inhibits IFN-λ1 production either at the step or upstream of TBK1 [53]. Foot-and-mouth disease virus (FMDV) leader proteinase suppresses the activation of the IFN-λ1 promoter by RIG-I, MDA5, IPS-1, IRF3, or IRF7 [54]. PDCoV inhibits the type III IFN response by targeting MAVS [27]. CSFV N protein inhibits IRF1 expression and its nuclear translocation [42]. Therefore, we investigated the potential target of nsp1 and found that SADS-CoV infection significantly reduced the activation of IFN-λ1 promoter mediated by MAVS, TBK1, IKKε, and IRF1, suggesting that nsp1 might target IRF1 to inhibit the type III IFN response.

Indeed, IRF1 is a critical component of the host antiviral defense system and plays a crucial role in inducing IFN-λ production [32]. Viruses, on the other hand, inhibit the nuclear translocation of IRF1 as a means to antagonize IFN-λ production [27, 43]. In this study, we discovered that SADS-CoV nsp1 blocked the nuclear translocation of IRF1 without directly interacting with it. Interestingly, viruses employ diverse mechanisms to regulate IRF1 activity at both the mRNA transcript and protein levels [35]. For example, the core structural protein of hepatitis C virus represses IRF1 synthesis at the transcription level [55]. Human immunodeficiency virus targets IRF1 for ubiquitination and proteasomal degradation to evade the IRF1-mediated host immune response [56]. PEDV nsp1 inhibits the nuclear translocation of IRF1 and reduces the number of peroxisomes to suppress IRF1-mediated induction of type III IFNs [43]. In our study, we observed that the increasing amount of SADS-CoV or nsp1 led to a gradual decrease in the level of endogenous IRF1 protein. We examined the effect of nsp1 on IRF1 transcription and found no significant variation in IRF1 mRNA levels in the presence of nsp1. Therefore, we speculated that nsp1 might regulate IRF1 at the protein level. This was confirmed by our experiments, which showed that nsp1 accelerated the degradation of IRF1 after treatment with CHX. Furthermore, treatment with MG132 or CQ demonstrated that nsp1 might degrade IRF1 via the proteasomal pathway.

In summary, our study reveals that SADS-CoV suppresses IFN-λ1 production, and nsp1 acts as a potent antagonist of IFN-λ1. SADS-CoV nsp1 blocks the nuclear translocation of IRF1 and targets IRF1 for ubiquitin-mediated proteasome degradation. These findings enhance our understanding of the immune evasion strategies employed by SADS-CoV and may provide insights for the development of future therapeutic approaches to combat SADS.

### Supplementary Information

**Additional file 1. Primers used for RT-qPCR.**

**Additional file 2. The inhibitory effect of porcine IFN-λ1 on SADS-CoV in IPI-FX cells detected by IFA (A), RT-qPCR (B), and Western Blot (C).**

## Data Availability

The datasets generated during the current study are available from the corresponding author upon reasonable request.
