# Supplementary material for: Swine acute diarrhea syndrome coronavirus Nsp1 suppresses IFN-λ1 production by degrading IRF1 via ubiquitin–proteasome pathway
Source: Vet Res. 2024 Apr 8;55:45. doi: 10.1186/s13567-024-01299-6 (PMC11003034; doi:10.1186/s13567-024-01299-6)
Supplement: Supplementary file 1 — Additional file 1. Primers used for RT-qPCR. [file 13567_2024_1299_MOESM1_ESM.docx]

**Additional file 1. Primers used for RT-qPCR**

| **Primers** | **Sequence (5’-3’)** |
| --- | --- |
| IFN-λ1-F | GGTGCTGGCGACTGTGATG |
| IFN-λ1-R | GATTGGAACTGGCCCATGTG |
| IFN-λ3-F | ACTTGGCCCAGTTCAAGTCT |
| IFN-λ3-R | CATCCTTGGCCCTCTTGA |
| IFN-λ4-F | GTCCGTGTCTGCCTTGTCACTGAC |
| IFN-λ4-R | AGGACTTTGGGCCCTTTCACTATGC |
| IRF1-F | GCAGGACTTGGACATTGAACAGGCC |
| IRF1-R | CTCAGTTAATTTCCCCTCCTCGTCCTCATC |
| SADS-CoV-N-F | CTGACTGTTGTTGAGGTTAC |
| SADS-CoV-N-R | TCTGCCAAAGCTTGTTTAAC |
| GAPDH-F | CCTTCCGTGTCCCTACTGCCAAC |
| GAPDH-R | GACGCCTGCTTCACCACCTTCT |
