# Supplementary material for: Swine acute diarrhea syndrome coronavirus Nsp1 suppresses IFN-λ1 production by degrading IRF1 via ubiquitin–proteasome pathway
Source: Vet Res. 2024 Apr 8;55:45. doi: 10.1186/s13567-024-01299-6 (PMC11003034; doi:10.1186/s13567-024-01299-6)

**Additional file 2. The inhibitory effect of porcine IFN-λ1 on SADS-CoV in IPI-FX cells detected by IFA (A), RT-qPCR (B), and Western Blot (C).**


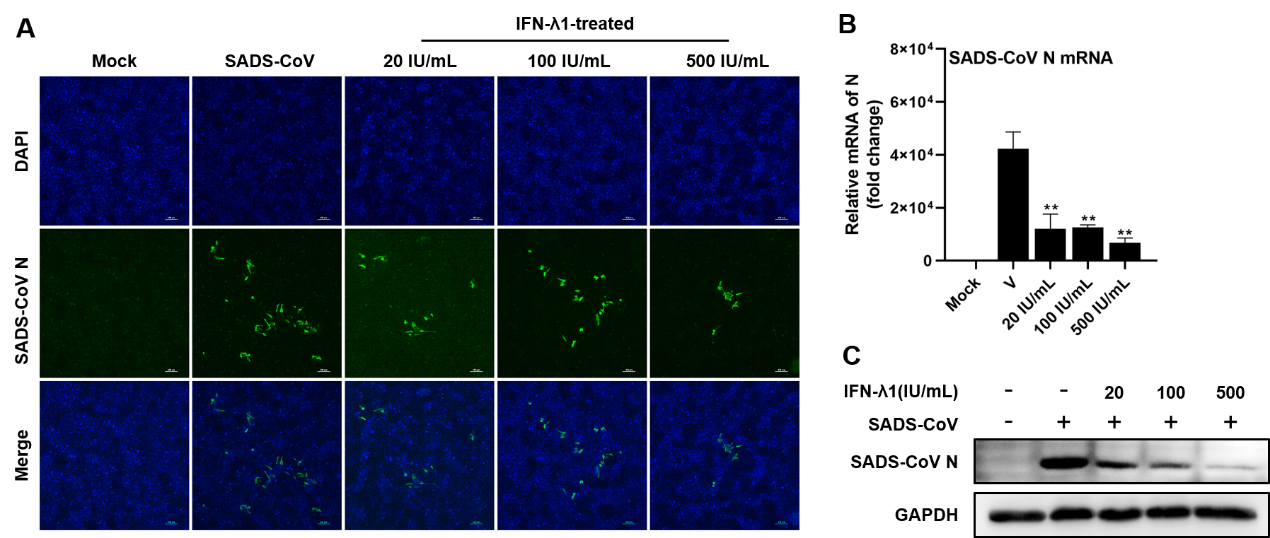

Supplement: Supplementary file 2 — Additional file 2. The inhibitory effect of porcine IFN-λ1 on SADS-CoV in IPI-FX cells detected by IFA (A), RT-qPCR (B), and Western Blot (C). [file 13567_2024_1299_MOESM2_ESM.docx]
